# Visual Mapping of Global Studies on Dental Hygienists: Trends and Emerging Hotspots

**DOI:** 10.3290/j.ohpd.c_2269

**Published:** 2025-09-11

**Authors:** Xueli Wan, Xiaorong Zhou, Wenqiang Jiang, Yifan Shen, Yifei Du, Yongle Shi

**Affiliations:** a Xueli Wan Researcher, State Key Laboratory of Oral Diseases & National Center for Stomatology & National Clinical Research Center for Oral Diseases, Post-Anesthesia Care Unit, West China Hospital of Stomatology, Sichuan University, Chengdu Sichuan, 610041, China. Study concept and design, conducted the research, collated the data, and wrote the manuscript.; b Xiaorong Zhou Researcher, State Key Laboratory of Oral Diseases & National Center for Stomatology & National Clinical Research Center for Oral Diseases, Emergency Department, West China Hospital of Stomatology, Sichuan University, Chengdu Sichuan, 610041, China. Study concept and design, conducted the research, collated the data, and wrote the manuscript.; c Wenqiang Jiang Researcher, State Key Laboratory of Oral Diseases & National Center for Stomatology & National Clinical Research Center for Oral Diseases, Emergency Department, West China Hospital of Stomatology, Sichuan University, Chengdu Sichuan, 610041, China. Study concept and design, conducted the research, collated the data, and wrote the manuscript.; d Yifan Shen Researcher, State Key Laboratory of Oral Diseases & National Center for Stomatology & National Clinical Research Center for Oral Diseases, Emergency Department, West China Hospital of Stomatology, Sichuan University, Chengdu Sichuan, 610041, China. Study concept and design, conducted the research, collated the data, and wrote the manuscript.; e Yifei Du Researcher, State Key Laboratory of Oral Diseases & National Center for Stomatology & National Clinical Research Center for Oral Diseases, Post-Anesthesia Care Unit, West China Hospital of Stomatology, Sichuan University, Chengdu Sichuan, 610041, China. Study concept and design, conducted the research, collated the data, and wrote the manuscript.; f Yongle Shi Researcher, State Key Laboratory of Oral Diseases & National Center for Stomatology & National Clinical Research Center for Oral Diseases, Emergency Department, West China Hospital of Stomatology, Sichuan University, Chengdu Sichuan, 610041, China. Study concept and design, conducted the research, collated the data, and wrote the manuscript.

**Keywords:** dental hygienists, dental hygiene education, emerging research trends, visualisation analysis

## Abstract

**Purpose:**

To analyse the current status and emerging research trends in the field of dental hygienists using a bibliometric analysis.

**Methods:**

Relevant literature on dental hygienists published up to 28 January 2025, was retrieved from the Web of Science Core Collection database. Bibliometric and visualisation analyses were conducted using Bibliometrix, VOSviewer, CiteSpace, Pajek, Tableau and Excel.

**Results:**

A total of 1,835 publications related to dental hygienists were included. The annual number of publications showed a fluctuating upward trend, peaking at 167 articles in 2024. The research involved 94 countries and regions, as well as 209 institutions. The United States had the highest publication output, with the University of Michigan ranking as the most prolific institution. The top three high-frequency keywords were ‘dental hygienists’ (323 occurrences), ‘oral health’ (275), and ‘dental hygiene’ (260). Keyword clustering analysis identified 13 thematic clusters. The most prominent emerging keyword was ‘allied dental education’. Notably, ‘infection control’ and ‘health’ started to gain prominence in 2022.

**Conclusion:**

Global dental hygiene research centres in North America and Europe, led by US institutions, with evolving priorities from prevention to public health integration, particularly in infection control. These findings inform tailored strategies for developing countries to advance DH professionalisation and address oral health disparities.

The global rise in oral disease burden, along with a rapidly ageing population,^29^ has drawn growing attention to dental hygienists (DHs) as a critical workforce in preventive oral healthcare. Their professional development is increasingly viewed as a key issue in health system reform, particularly as countries seek to expand access to cost-effective and non-invasive care.

The DH profession originated in the early twentieth century in the United States, where pioneers such as Alfred Fones helped transform the role from supporting nursing function into an independent profession grounded in preventive medicine.^11^

Today, many high-income countries, especially in North America and Europe, have established a well-defined professional system for DHs, supported by higher education pathways and formal legal recognition. In the United States, DHs are required to complete a two-year associate degree and pass the National Board Written Examination (NBDHE) along with state-level clinical exams.^4,27^ In the United Kingdom, the qualification process included a three-year undergraduate degree and registration with the General Dental Council (GDC).^12^ In several countries, such as Sweden, Canada, the Netherlands, and certain US states, DHs are legally authorised to independently carry out periodontal assessments, perform non-surgical periodontal therapy, and deliver oral health education.^21,28,31^ Such changes indicate a notable progression in the profession toward greater recognition, responsibility, and independence.

In contrast, many Asian countries or regions have seen more limited progress towards full professionalisation. In Japan, South Korea, and Thailand, DHs typically undergo 2–3 years of vocational training and pass national certification exams. However, their scope of practice is often narrowly defined by law, limited to basic, task-oriented support for dentists, such as preparing instruments or performing routine cleanings, with no legal authority to practice independently or carry out more advanced procedures.^25,34^

Although the Healthy China 2030 blueprint explicitly calls for improved oral health management, in China there is currently no national certification standard or unified education system for DHs.^18,33^ Most existing training is short-term or offered internally by hospitals, lacking the formal academic structure and standardisation seen in Western systems.^19^ This underdevelopment of the profession mirrors and is further perpetuated by disparities in global research production related to DHs.

To better understand these patterns, this study conducts a comprehensive bibliometric analysis of 1,835 publications related to DHs, indexed in the Web of Science Core Collection database up to January 2025. Using analytical tools including Bibliometrix, VOSviewer, CiteSpace, Pajek, Tableau, and Excel, we aim to address the following research questions:

**Collaboration networks:** Which countries, regions, and institutions serve as key hubs in the global DH research landscape? What spatial patterns define international research collaboration?
**Thematic evolution:** How have research priorities in the field of DHs shifted over time? What are the emerging frontiers?
**Policy implications:** What lessons from global experience can inform the development of DH professionalisation in countries where the role remains underdeveloped?

## MATERIALS AND METHODS

### Search Strategy

This study employed a quantitative bibliometric approach, guided by the theoretical framework of science mapping.^6^ Adhering to standard bibliometric analysis procedures, we systematically conducted the following steps: data collection and screening, publication trend analysis, collaboration network mapping, keyword co-occurrence and clustering analysis, and burst detection. These analyses were conducted to elucidate the structure, evolution, and current research frontiers within this field. This study retrieved literature related to DHs from the Web of Science Core Collection (WoSCC). Advanced search was conducted using the following topic terms: ‘dental hygienist’; ‘dental hygienists’; ‘dental hygiene education’; ‘the scope of dental hygienists’ practice’; and ‘dental hygiene programs’. The search covered all records from database inception to 28 January 2025, and was limited to English-language articles and reviews.

### Eligibility Criteria

The inclusion criteria were as follows: (1) publications with a central focus on DHs; (2) article types including clinical trials, case reports, animal studies, literature-based research, and reviews; (3) complete bibliographic information available, including titles, authors, keywords, sources, etc; and (4) written in English.

The exclusion criteria were as follows: (1) publications with incomplete metadata or duplicate entries; (2) letters, conference abstracts, book chapters, monographs, or retracted studies; and (3) publications not directly related to the topic of DHs.

### Data Processing

**Fig 1:**
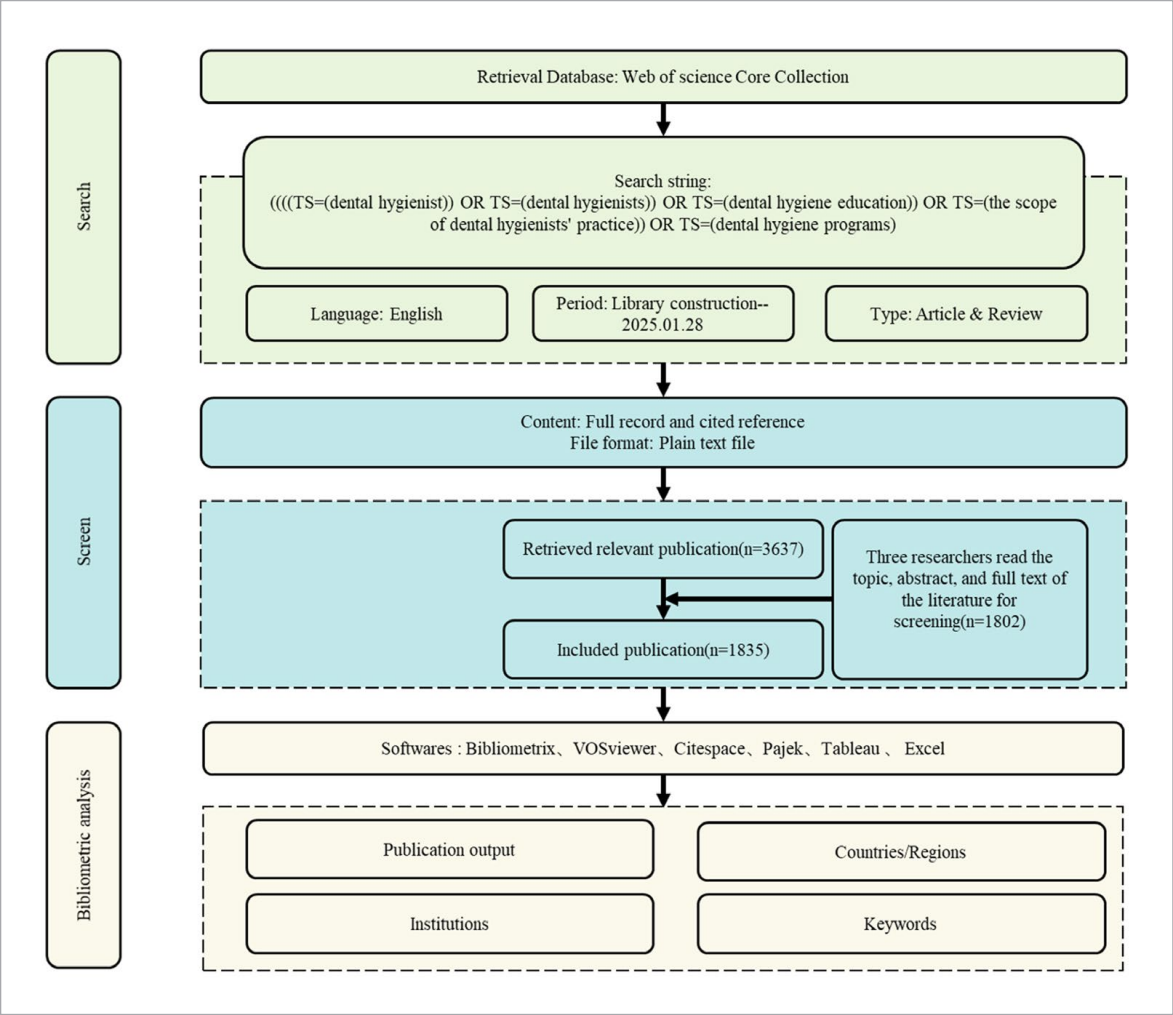
Flowchart of the literature screening and selection process.

Three independent researchers screened the titles, abstracts, and full texts of the retrieved articles. In cases of disagreement, final inclusion decisions were made by an expert in the field. A total of 1,835 articles were ultimately included. The detailed search flow is presented in Figure 1.

### Data Analysis

#### Annual publication trend analysis

We used Excel to analyse the annual distribution of publications from January 1997 to January 2025. Polynomial curve fitting was applied through exponential regression to model the publication growth trend. A one-year time slice was used.

#### Bibliometric analysis

National and regional research trends were visualised using the Bibliometrix package in R Studio (version 4.2.2), in combination with Tableau. Institutional collaboration networks were mapped using VOSviewer (version 1.6.18) and further refined with Pajek. Keyword co-occurrence and clustering analyses were performed using CiteSpace (version 6.2. R3).

### Research Ethics

All data analysed in this study were obtained from publicly available databases. Therefore, ethics committee approval was not required.

## RESULTS

### Annual Publication Trends

From 1997 to 2025, the number of publications on DHs showed an overall upward trend with noticeable fluctuations. With a polynomial curve fitting score (R²) of 0.607, the model indicates that the number of articles has increased over time. Based on publication volume, the development of DH-related research can be divided into three distinct phases:

**Phase 1:** Slow growth (1997–2005). During this period, the number of publications increased slowly from 20 in 1997 to 25 in 2005. A total of 174 articles were published, accounting for 9% of all included studies.
**Phase 2:** Rapid growth (2006–2018). Starting in 2006, publication output grew markedly, reaching 109 articles by 2018.
**Phase 3:** Stable and accelerated expansion (2019–2025). Research activity remained consistently high, peaking at 167 publications in 2024.

**Fig 2:**
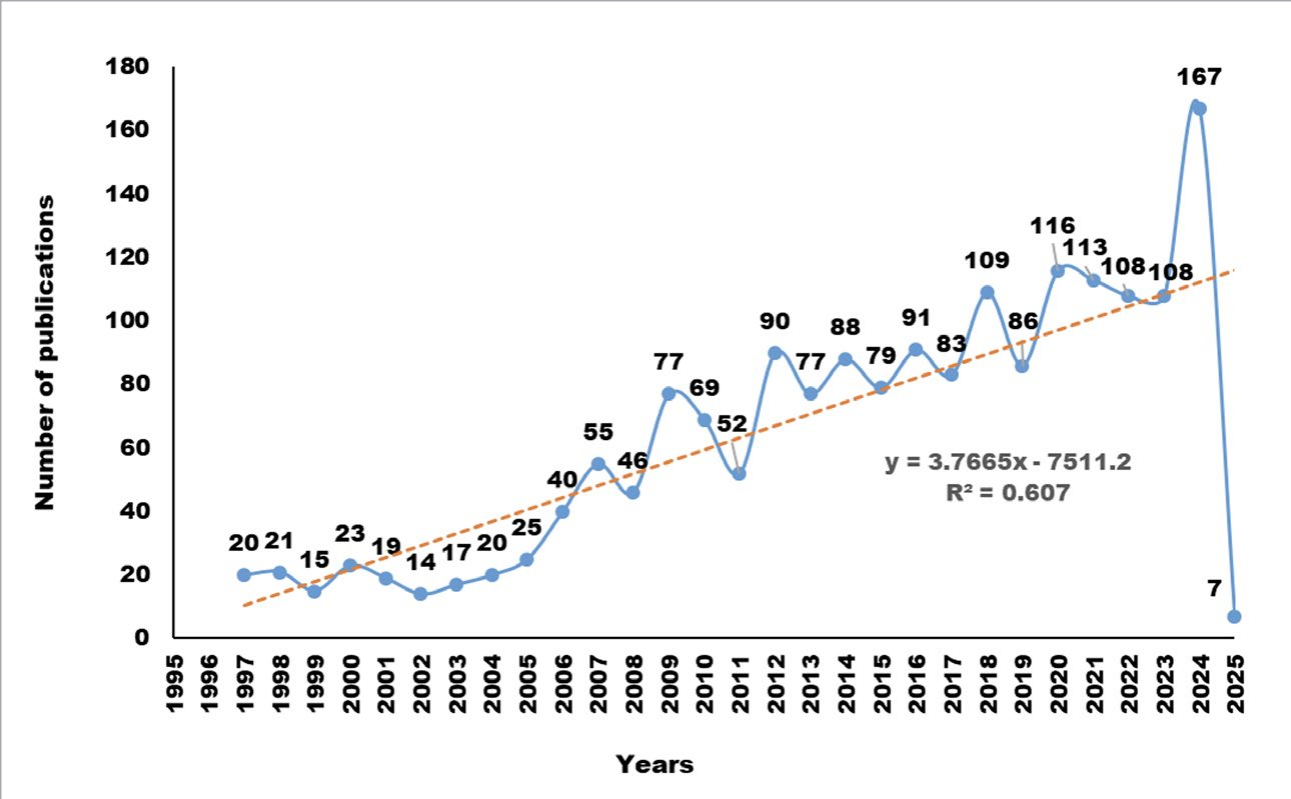
Annual global publication trends related to DHs (1997–2025).

These trends indicate a sustained and growing academic interest in the professional development and evolving role of DHs over the past two decades (Fig 2).

### Country/Region and Institutional Contributions

**Fig 3a to c:**
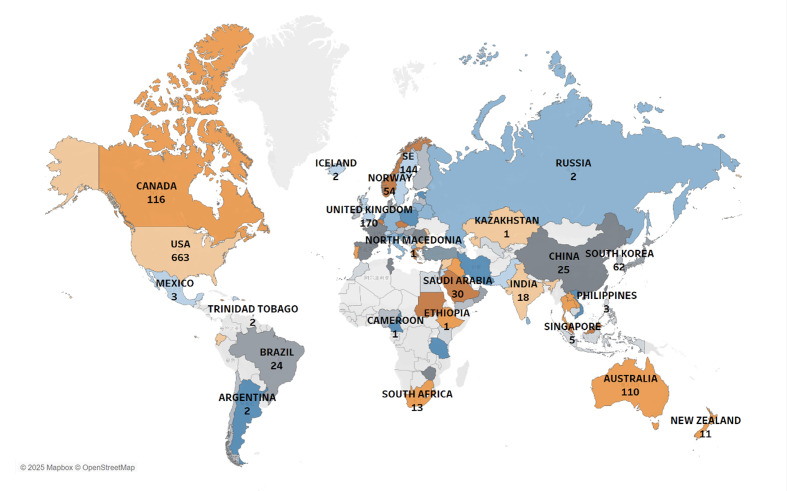

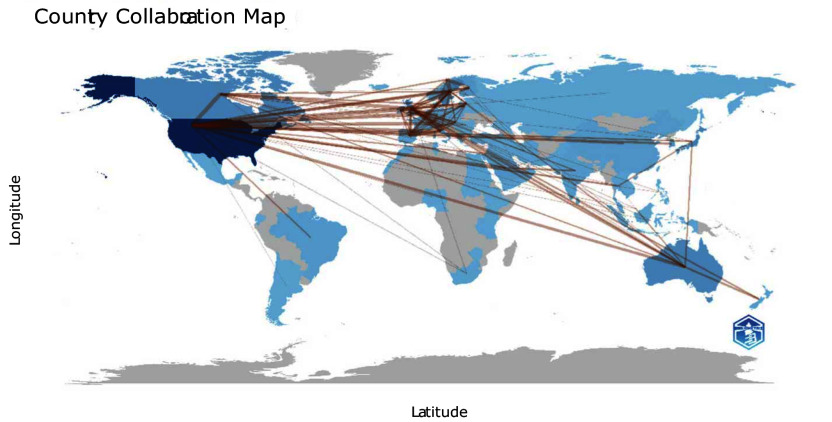

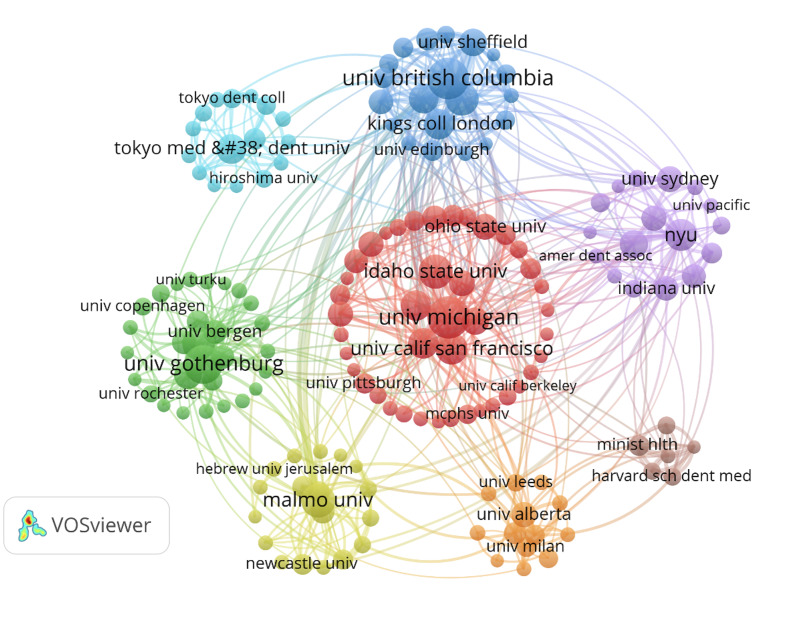
Country/region and institutional contributions. (a) Publication output by country/region, (b) international collaboration network, and (c) institutional collaboration network.

A total of 94 countries or regions and 1,883 institutions contributed to the body of research on DHs. The United States emerged as the leading contributor, with 663 publications, followed by the United Kingdom (170 articles) and Sweden (144 articles) (Fig 3a).

In the international collaboration network, the United States serves as a central hub, with a dense network of connections extending across all continents, including North America, South America, Europe, Asia, Africa, and Oceania, indicating its strong collaborative ties (Fig 3b). Collaborations among Western countries, particularly in Europe and North America, were notably strong and often served as key channels for facilitating broader international research cooperation. At the institutional level, the University of Michigan (USA) ranked first with 48 publications, followed by the University of British Columbia (Canada) with 46, and the University of Gothenburg (Sweden) with 40.

The global institutional collaboration network formed eight major clusters, reflecting distinct research alliances across regions (Fig 3c).

### Keyword Analysis

#### Co-occurrence analysis of keywords

The keyword co-occurrence network provides a concise and structured summary of the core research themes in a given field. Higher keyword frequencies indicate greater research attention, while co-occurrence patterns reveal prevailing hotspots and the overall landscape of scholarly activity.

**Fig 4:**
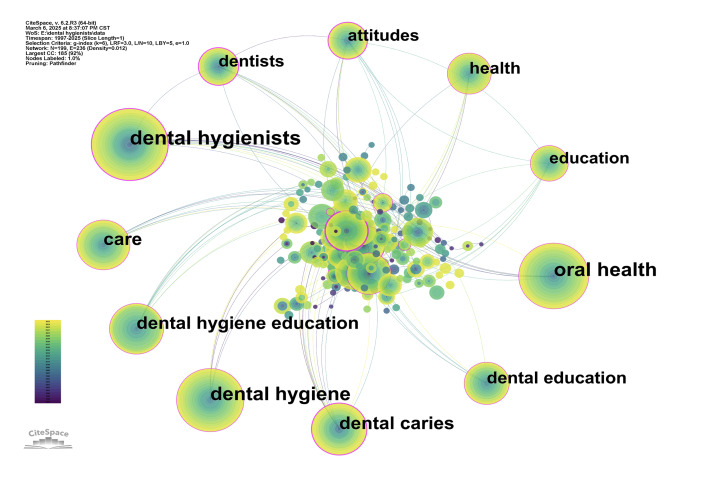
Top 11 most frequently occurring keywords in DH research.

Table 1 summarises the top 10 most frequently occurring keywords and those with the highest betweenness centrality. Dental hygienists, oral health, and dental hygiene emerged as the most frequently used terms, indicating their central importance within the field. In contrast, disease, dental caries, and attitudes ranked highest in betweenness centrality, suggesting their function as conceptual bridges across diverse research areas (Fig 4).

**Table 1:** Top 10 most frequently occurring keywords and keywords with the highest betweenness centrality in DH research

Rank	Top keywords by frequency	Frequency	Centrality	Top keywords by betweenness centrality	Frequency	Centrality
1	dental hygienists	323	0.29	disease	93	0.41
2	oral health	275	0.14	dental caries	173	0.38
3	dental hygiene	260	0.12	attitudes	106	0.36
4	dental caries	173	0.38	dentists	109	0.35
5	care	170	0.13	children	93	0.33
6	dental hygiene education	164	0.16	dental hygienists	323	0.29
7	dental education	118	0.14	preventive dentistry	6	0.29
8	health	115	0.13	adolescents	30	0.25
9	dentists	109	0.35	cary	84	0.19
10	attitudes	106	0.36	dental hygiene education	164	0.16


#### Keyword clustering analysis

Keyword clustering reveals the thematic structure of research within the field. Based on the log-likelihood ratio (LLR) algorithm, keywords were grouped into 13 distinct clusters. The modularity value (Q = 0.572; >0.3) and the mean silhouette score (S = 0.9235; >0.7) indicate a well-structured and reliable clustering result 6.

Among the clusters, Cluster #0 (dental hygienist) had the highest silhouette score and the largest number of nodes, suggesting that this theme is the dominant research focus in the field. Table 2 lists the clustering results, including cluster number, silhouette score, number of nodes, label (LLR-based), and average publication year.

**Table 2:** Keyword clustering based on the LLR algorithm

Cluster	Silhouette score	Number of nodes	Label (LLR)	Average year
0	0.990	24	dental hygienist	2009
1	0.982	22	dental caries	2008
2	0.887	22	dental hygiene education	2011
3	0.961	15	oral health	2011
4	0.991	14	musculoskeletal disorders	2007
5	0.902	14	oral hygiene	2009
6	0.846	14	cultural diversity	2010
7	0.940	13	oral cancer	2009
8	0.834	13	hygienist therapists	2012
9	0.813	13	dental hygiene	2006
10	0.900	10	independent practice	2007
11	0.943	6	dental auxiliaries	2010
12	0.985	5	mental health	2022


#### Keyword timeline and time zone analysis

Keyword timeline analysis provides a temporal overview of how research themes have evolved. It illustrates the historical development of core topics and highlights periods of peak attention within each cluster.

Clusters such as #1 (dental caries) and #9 (dental hygiene) exhibited broad time spans, with active research spanning nearly the entire period from 1997 to 2024. In contrast, Cluster #12 (mental health) represents a more recent research frontier, with active concentration between 2017 and 2024.

**Fig 5a to c:**
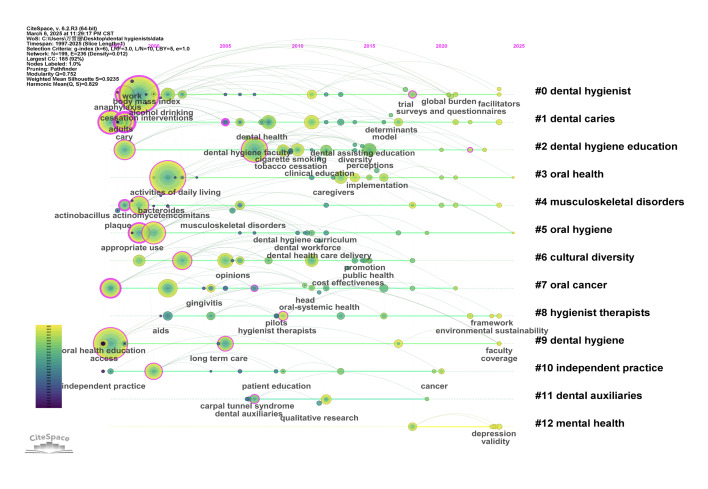

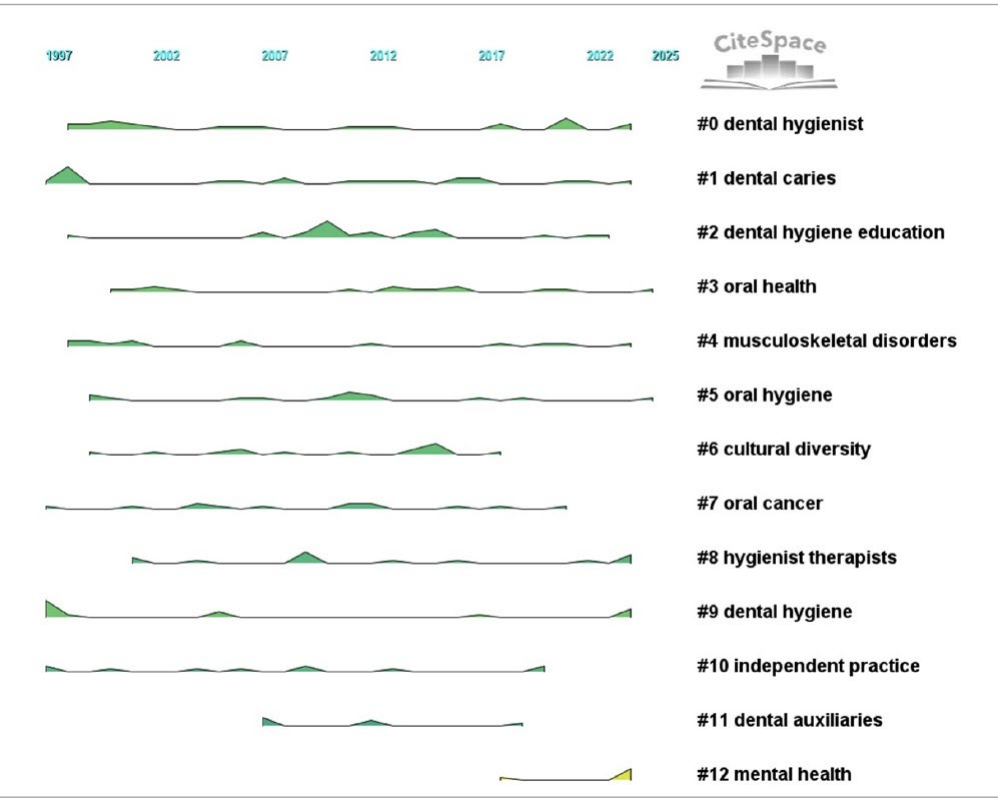

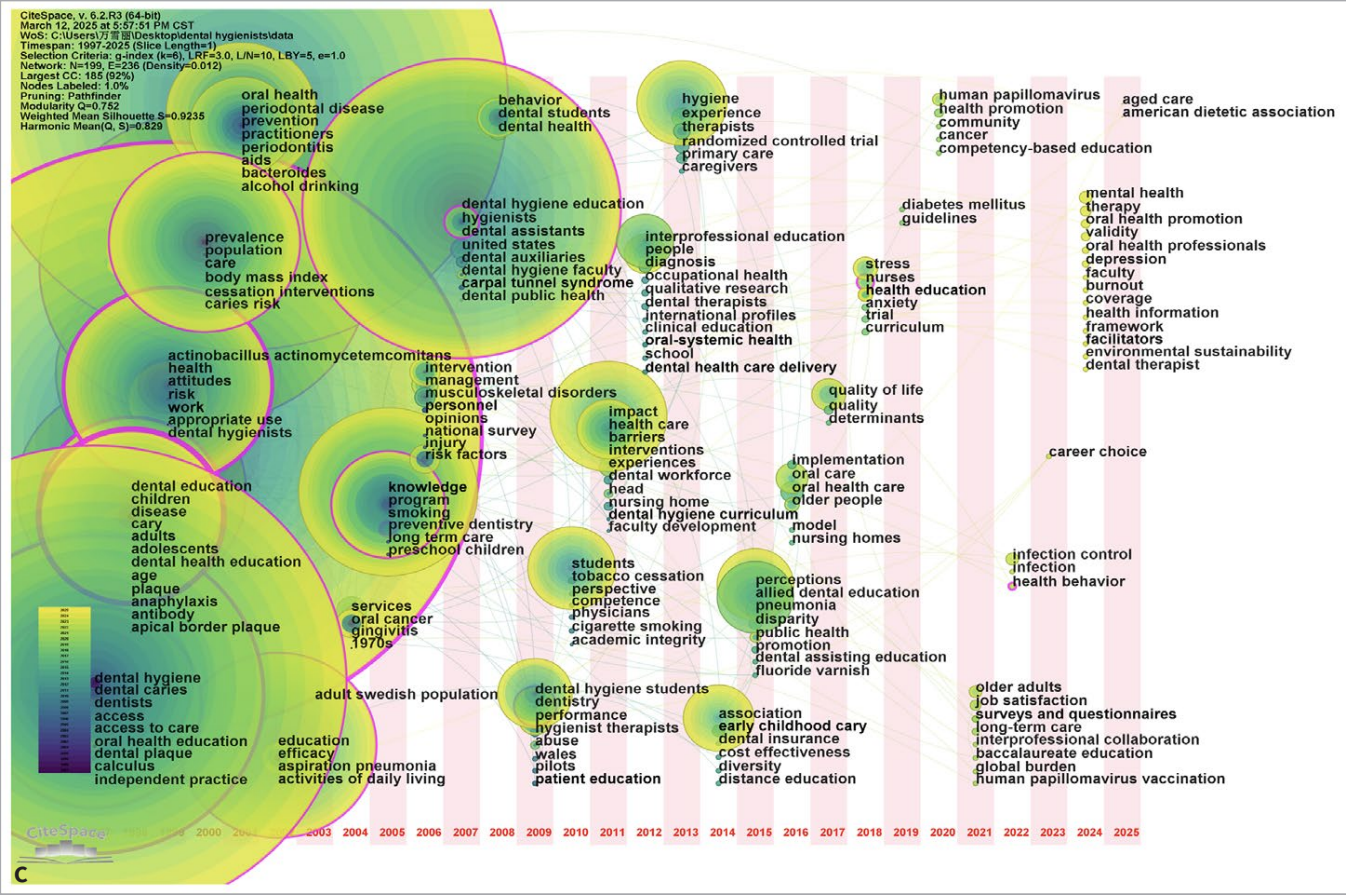
Keyword timeline and time zone analysis. (a) Keyword timeline map, (b) keyword burst diagram, and (c) keyword time zone visualisation

Figure 5a displays the keyword timeline map by cluster, while Figure 5b presents the keyword burst map, highlighting periods of intense interest. Figure 5c shows representative high-frequency keywords by publication year.

#### Keyword bursts analysis

**Fig 6:**
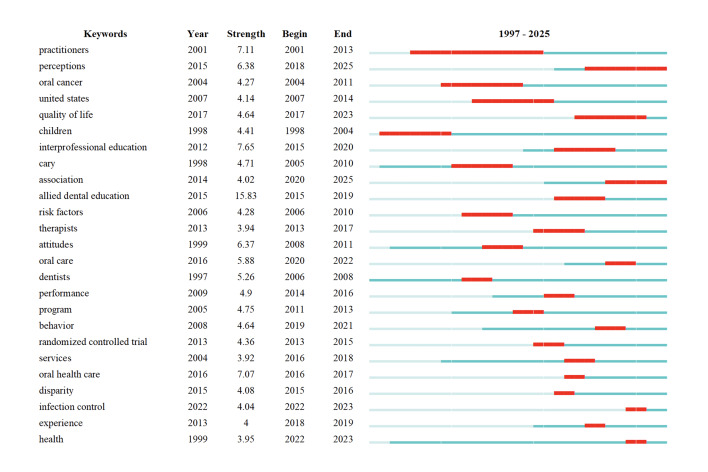
Top 25 keywords with the strongest citation bursts in DH research.

Figure 6 displays the top 25 keywords with the strongest citation bursts in the field of DHs. The keyword ‘allied dental education’ showed the highest burst intensity, with a score of 15.83, indicating a sharp rise in research attention over a specific period.

The keyword ‘practitioners’ exhibited the longest duration of sustained attention, spanning from 2001 to 2013, suggesting its enduring relevance during that time. Notably, in 2022, the keywords ‘infection control’ and ‘health’ emerged as new burst terms, implying their potential to become major research hotspots in the coming years.

## DISCUSSION

This study conducted a comprehensive bibliometric and visual analysis of research on DHs, identifying international collaboration patterns, key research themes, and their evolution over time. A general upward trend in publication volume can be attributed to increased global awareness of public and oral health, accelerated population ageing, national policy initiatives and advances in digital technologies.

### Collaborative Networks: Core Hubs and International Research Patterns

Our analysis identified clear geographic and institutional patterns in global DHs research. High-output countries were primarily concentrated in Europe and North America, with the United States, the United Kingdom, and Sweden leading the field. These countries not only produced the highest number of publications but also housed the most influential institutions, namely the University of Michigan, the University of British Columbia, and the University of Gothenburg.

The dominance of the United States may reflect its well-established DHs education system, robust research infrastructure, and long-standing investment in oral health.^2,13^ The United Kingdom and Sweden followed closely, further highlighting the central role of Western countries in advancing DH research and shaping international academic influence.^9,10,17,24^ Collaboration within and between European and North American countries was particularly strong, creating a dense cooperation network that has extended its impact to other regions worldwide.

As global interest in oral health increases, more countries are participating in DH research, strengthening international academic exchange and contributing to the advancement of global oral health.

At the institutional level, the University of Michigan emphasises a prevention-oriented approach that integrates theory and practice, focusing on oral health education, community-based dental care, communication skills, and public health policy.^1,7,15,30^ The University of British Columbia focuses on oral health, dental hygiene education, periodontology, and service delivery, aiming to train professionals equipped to solve real-world problems.^3,32,35^ The University of Gothenburg is known for integrating DH research into public health systems, with an emphasis on disease prevention, clinical care, oral health education, and health policy.^8,22,23^

### Thematic Evolution: Shifting Research Hotspots and Emerging Frontiers

Keyword analysis revealed that the core research themes remain centred on the role of DHs, oral health management, and dental hygiene education, as reflected by the most frequent keywords ‘dental hygienists’, ‘oral health’, and ‘dental hygiene’. These themes indicate the continued importance of DHs in oral disease prevention and public health.

Based on keyword clustering, four major thematic areas were identified: (1) scope of practice (clusters #1, #3, #5, #7, #13); (2) oral healthcare delivery (clusters #0, #8, #9, #10, #11); (3) training models for dental hygiene (clusters #2, #6, #13); (4) occupational health of DHs (cluster #4, #13).

From a temporal perspective, research development can be divided into three major phases. From 1997 to 2010, the foundational phase took place. Focused on basic disease studies, early exploration of preventive measures, and the establishment of an educational system. Keywords included dental caries, dental hygiene education, and preventive dentistry. Between 2011 and 2020, integration with the public health phase occurred, which emphasised interdisciplinary collaboration, expanded professional roles, and implementation of public health policy. Notable keywords included ‘interprofessional education’, ‘diabetes mellitus’, ‘competency-based education’, and ‘healthcare delivery’. From 2021 to 2025, the Global Challenges and Technological Innovation phase took place, addressing global health issues, doctors’ mental well-being, artificial intelligence, and sustainable development. Keywords such as artificial intelligence, burnout, global burden, and environmental sustainability emerged during this period.

This progression illustrates a shift from localised disease management to an integrated global health ecosystem, with DHs playing a critical role throughout. Notable empirical studies have further emphasised this shift. For example, Boer et al^5^ conducted qualitative interviews with general dentists and DHs in a Dutch dental chain, identifying key factors that influenced collaboration, shared goals, leadership style, task distribution, and formalisation. Their findings highlight the importance of leadership and shared vision in enhancing interprofessional cooperation and improving care quality.

Similarly, Hama et al^16^ conducted a one-year prospective multicentre cohort study of 504 residents (mean age 87.4 years) in Japanese long-term care facilities. They found that those who received professional oral health management (OHM) from DHs had a significantly lower incidence of pneumonia than those who did not, highlighting the critical role of DHs in maintaining both oral and systemic health in elderly populations.

Emerging research frontiers are also gaining momentum. The appearance of ‘infection control’ and ‘health’ as burst keywords in 2022 suggests growing attention to the intersection of oral and public health. Additionally, the prominence of interprofessional education and allied dental education points to a future emphasis on team-based approaches and collaborative care models.

### Policy Implications: Lessons for Countries Without DHs Professionalisation

The findings from this study offer valuable insights for countries where DHs’ professionalisation is not yet well-established. For instance, Asian countries such as India and Thailand could adopt a phased approach to reform. In the long term, international partnerships could facilitate remote training programmes using Western curricula to strengthen DHs’ competencies in early disease detection. In the short term, Japan’s ‘oral hygiene therapist’ model could serve as a blueprint for establishing three-year diploma programmes along with national certification exams, prioritising deployment in underserved rural areas.^26^

In African countries, a more pragmatic approach should be appropriate, focusing on low-cost technology adaptation and task shifting.^14^ For example, partnerships with institutions such as the University of Gothenburg could support research on training nurses to assume basic DH roles after short-term certification, helping alleviate workforce shortages.

China necessitates an integrated reform mode that links policy, education, and practice. In terms of policy, the Swedish experience presents a paradigmatic example by integrating DHs into national public health programmes and clearly defining their roles in chronic disease management, supported by legislative protections.^20^ In education, the US example of dual-track clinical and research training should be adopted through international collaboration and curriculum accreditation. Pilot programmes should be launched in regions such as the Yangtze River Delta and Greater Bay Area, where DHs work alongside family doctors using AI-assisted diagnostic tools to improve service efficiency at the primary care level.

The success of core institutions in Europe and North America stems from a closed-loop ecosystem of linking research, education, and policy. For China and other developing countries, localised strategies are essential. China should leverage policy reform and international partnerships. Asian countries should focus on tiered professionalisation pathways, while African nations may prioritise access to essential services. These differentiated strategies can support the sustainable development of DH systems and help narrow global gaps in oral health equity.

## CONCLUSIONS

This study applied six bibliometric tools to analyse 1,835 publications on DHs, examining trends in publication volume, collaborative networks, research hotspots, and emerging directions. The results showed a steady global increase in DH-related research, suggesting the growing importance of DHs in promoting oral health and contributing to public health systems worldwide.

This study identifies Europe and North America, with the US as a primary contributor and key institutions such as the University of Michigan serving as central hubs, forming the core of global DH collaboration. Research priorities have evolved from foundational prevention to public health integration, now emphasising global challenges, technology, and mental well-being, with infection control emerging as critically important. To advance DH professionalisation and reduce global oral health inequities, tailored regional strategies are imperative: policy reforms and international partnerships in China, tiered developmental pathways across Asia, and improved healthcare access in Africa.

The results presented in this study can serve as a valuable reference for countries where the professionalisation of DHs is still in its early stages. By drawing on global research patterns and policy experiences, these countries can explore context-appropriate reforms to support the development of the DHs workforce.

However, it is important to note that this study is based solely on bibliometric data and lacks validation through clinical or practical outcomes. Future research, particularly in countries without an established DHs system, should consider applying these findings in clinical settings to assess their practical relevance and impact.

### Acknowledgements

This work was supported by the Clinical Research Program of West China Hospital of Stomatology, Sichuan University [LCYJ-HL-202303]. The authors declare no competing interests.
